# Violence motivated by perception of sexual orientation and gender identity: a systematic review

**DOI:** 10.2471/BLT.17.197251

**Published:** 2017-11-23

**Authors:** Karel Blondeel, Sofia de Vasconcelos, Claudia García-Moreno, Rob Stephenson, Marleen Temmerman, Igor Toskin

**Affiliations:** a Faculty of Medicine and Health Sciences, Ghent University, Campus UZ Gent, Building K3, 3rd floor, De Pintelaan 185, 9000 Gent, Belgium.; b Department of Reproductive Health and Research, World Health Organization, Geneva, Switzerland.; c Department of Health Behavior and Biological Sciences, School of Nursing, University of Michigan, Ann Arbor, United States of America.

## Abstract

**Objective:**

To assess the prevalence of physical and sexual violence motivated by perception of sexual orientation and gender identity in sexual and gender minorities.

**Methods:**

We searched nine databases without language restrictions for peer-reviewed and grey literature published from 2000 to April 2016. We included studies with more than 50 participants that measured the prevalence of physical and sexual violence perceived as being motivated by sexual orientation and gender identity or gender expression. We excluded intimate partner violence and self-harm. Due to heterogeneity and the absence of confidence intervals in most studies, we made no meta-analysis.

**Findings:**

We included 76 articles from 50 countries. These covered 74 studies conducted between 1995 and 2014, including a total of 202 607 sexual and gender minority participants. The quality of data was relatively poor due to a lack of standardized measures and sometimes small and non-randomized samples. In studies where all sexual and gender minorities were analysed as one population, the prevalence of physical and sexual violence ranged from 6% (in a study including 240 people) to 25% (49/196 people) and 5.6% (28/504) to 11.4% (55/484), respectively. For transgender people the prevalence ranged from 11.8% (of a subsample of 34 people) to 68.2% (75/110) and 7.0% (in a study including 255 people) to 49.1% (54/110).

**Conclusion:**

More data are needed on the prevalence, risk factors and consequences of physical and sexual violence motivated by sexual orientation and gender identity in different geographical and cultural settings. National violence prevention policies and interventions should include sexual and gender minorities.

## Résumé

Objectif** Estimer la prévalence des violences physiques et sexuelles motivées par la perception de l'orientation sexuelle et de l'identité de genre dans les minorités sexuelles.**

Méthodes** Nous avons recherché dans neuf bases de données, sans restriction de langue, des documents soumis à comité de lecture ou non et publiés entre 2000 et avril 2016. Nous avons inclus les études de plus de 50 participants qui ont mesuré la prévalence des violences physiques et sexuelles perçues comme étant motivées par l'orientation sexuelle et l'identité ou l'expression de genre. Nous avons exclu les violences conjugales et les actes auto-agressifs. En raison de l'hétérogénéité des études et de l'absence d'intervalle de confiance dans la plupart d'entre elles, nous n'avons pas réalisé de méta-analyse.**

Résultats** Nous avons pris en compte 76 articles provenant de 50 pays. Ceux-ci s’intéressaient à 74 études, menées entre 1995 et 2014, qui portaient sur un total de 202 607 participants issus de minorités sexuelles. La qualité des données était relativement mauvaise à cause de l'absence de mesures standardisées et, parfois, d'échantillons réduits et non randomisés. Dans les études qui ont analysé l'ensemble des minorités sexuelles comme une seule population, la prévalence des violences physiques et sexuelles allait respectivement de 6% (dans une étude sur 240 personnes) à 25% (49/196 personnes) et de 5,6% (28/504) à 11,4% (55/484). Pour les personnes transgenre, la prévalence allait de 11,8% (dans un sous-groupe de 34 personnes) à 68,2% (75/110) et de 7,0% (dans une étude sur 255 personnes) à 49,1% (54/110).**

Conclusion** Il est nécessaire d'avoir davantage de données sur la prévalence, les facteurs de risque et les conséquences des violences physiques et sexuelles motivées par l'orientation sexuelle et l'identité de genre dans différentes régions et différents contextes culturels. Les politiques et les actions nationales de prévention de la violence devraient prendre en compte les minorités sexuelles.**

## Resumen

**Objetivo:**

Evaluar la prevalencia de la violencia física y sexual motivada por la percepción de la orientación sexual y la identidad de género en las minorías sexuales y de género.

**Métodos:**

Se realizaron búsquedas en nueve bases de datos sin restricciones de idioma en busca de literatura gris y examinada por expertos publicada desde 2000 hasta abril de 2016. Incluimos estudios con más de 50 participantes que midieron la prevalencia de la violencia física y sexual percibida como motivada por la orientación sexual y la identidad o la expresión de género. Excluimos la violencia en la pareja íntima y la autolesión. Debido a la heterogeneidad y la ausencia de intervalos de confianza en la mayoría de los estudios, no realizamos ningún metanálisis.

**Resultados:**

Se incluyeron 76 artículos de 50 países. Estos cubrieron 74 estudios realizados entre 1995 y 2014, incluyendo un total de 202.607 participantes de minorías sexuales y de género. La calidad de los datos fue relativamente baja debido a la falta de medidas estandarizadas y, en ocasiones, muestras pequeñas y no aleatorizadas. En los estudios en los que todas las minorías sexuales y de género se analizaron como una única población, la prevalencia de la violencia física y sexual varió del 6% (en un estudio que incluía a 240 personas) al 25% (49/196 personas) y del 5,6% (28/504) al 11,4% (55/484), respectivamente. Para las personas transexuales, la prevalencia varió del 11,8% (de una submuestra de 34 personas) al 68,2% (75/110) y del 7,0% (en un estudio que incluyó a 255 personas) al 49,1% (54/110).

**Conclusión:**

Se necesitan más datos sobre la prevalencia, los factores de riesgo y las consecuencias de la violencia física y sexual motivada por la orientación sexual y la identidad de género en diferentes entornos geográficos y culturales. Las políticas e intervenciones nacionales de prevención de la violencia deberían incluir las minorías sexuales y de género.

## ملخص

**الغرض:**

تقييم مدى انتشار العنف الجسدي والجنسي المدفوع بمفهوم الميل الجنسي والهوية الجنسانية في الأقليات الجنسية والجنسانية.

**الطريقة:**

بحثنا في تسع قواعد للبيانات دون التقيد باللغة عن الكتابات غير الرسمية والكتابات التي خضعت للاستعراض الندّي والمنشورة منذ عام 2000 حتى أبريل/نيسان 2016. وشمل البحث دراسات ضمت أكثر من 50 مشاركًا لقياس مدى انتشار العنف الجسدي والجنسي الذي يُنظر إليه على ناتج عن الميل الجنسي والهوية الجنسانية أو السلوك المعبر عن الهوية الجنسانية. واستبعدنا حالات عنف العشير وإيذاء النفس. ونظرًا لعدم التجانس وافتقار معظم الدراسات إلى النسب الأرجحية، فلم نعمل على إجراء تحليل تلوي.

**النتائج:**

ضم البحث الذي أجريناه 76 مقالة من 50 دولة. وشملت تلك المقالات 74 دراسة تم إجراؤها في الفترة بين عامي 1995 و2014، وضمت تلك الدراسات عددًا من المشاركين الذين ينتمون إلى أقليات جنسية وجنسانية يبلغ 202607 إجمالاً. وكانت نوعية البيانات منخفضة المستوى نسبيًا نظرًا لغياب الإجراءات الخاضعة لمعيار موحد وانخفاض حجم العينات أو الاعتماد على عينات غير عشوائية في بعض الأحيان. وسجلت الدراسات التي خضعت فيها جميع الأقليات الجنسية والجنسانية للتحليل باعتبارها تمثل مجموعة سكانية واحدة نسبةً لانتشار العنف الجنسي والجنساني تتراوح بين 6‏% (في دراسة اشتملت على 240 شخصًا) و‏‏25% (49/196 شخصًا) وبين ‏5.6‏% (‏28/504) و11.4‏% (‏55/484)، على التوالي. وتراوحت نسبة انتشار العنف فيما يتعلق بمغايري الهوية الجنسانية بين 11.8‏% (في عينة فرعية تتكون من 34 شخصًا) و68.2‏% (75/110) وكذلك بين 7‏% (في دراسة ضمت 255 شخصًا) و49.1‏% (54‏/110).

**الاستنتاج:**

يحتاج الأمر إلى توفر المزيد من البيانات بشأن مدى انتشار العنف الجسدي والجنسي بسبب مفهوم الميل الجنسي والهوية الجنسانية وعواقب ذلك العنف وعوامل الخطورة المرتبطة به، وذلك في بيئات جغرافية وثقافية مختلفة. ويجب أن تشتمل السياسات الوطنية والتدخلات الرامية لمنع العنف على إجراءات تتعلق بالأقليات الجنسية والجنسانية.

## 摘要

**目的:**

旨在评估性与性别认同少数群体中由于性取向和性别认同感知而引发的人身暴力和性暴力的盛行率。

**方法:**

我们针对 2000 年至 2016 年 4 月发表的同行评审和灰色文献，搜索了 9 个没有语言限制的数据库。我们纳入了超过 50 名参与者的研究，这些参与者被认为是由于性取向、性别认同或性别表达引发的人身暴力和性暴力。我们排除了亲密伴侣的暴力和自我伤害。由于大多数研究的异质性和置信区间的缺失，我们没有进行元分析。

**结果:**

我们收录了 50 个国家的 76 篇文章。这些研究涵盖了 1995 至 2014 年间进行的 74 项研究，其中包括总计 202 607 名性与性别认同少数参与者。由于缺乏标准化的措施，有时是小样本和非随机样本，因此数据质量相对较差。在研究中，所有性与性别认同少数群体作为一类整体来研究，人身暴力和性暴力的盛行率分别从 6%（一项包括 240 人的研究中）到 25%（196 人中 49 人）和 5.6% (28/504) 到 11.4% (55/484)。对于变性人来说，盛行率分别从 11.8%（34 人的子样本）到 68.2% (75/110) 和 7.0%（包含 255 人的研究）到 49.1% (54/110)。

**结论:**

需要更多有关不同地理和文化背景下由于性取向和性别认同感知而引发的人身暴力和性暴力的盛行率、风险因素和后果的数据。国家预防暴力的政策和干预措施应该将性与性别认同少数群体包括在内。

## Резюме

**Цель:**

Оценить распространенность физического и сексуального насилия на почве сексуальной ориентации и гендерной индивидуальности в отношении сексуальных и гендерных меньшинств.

**Методы:**

В девяти базах данных авторы провели поиск без языковых ограничений «серой» и рецензируемой специалистами литературы, опубликованной с 2000 года по апрель 2016 года. В анализ были включены исследования (в них приняли участие более 50 респондентов), посвященные оценке распространенности физического и сексуального насилия на почве сексуальной ориентации, гендерной индивидуальности или гендерного самовыражения. Исключалось насилие со стороны полового партнера и причинение вреда самому себе. Из-за неоднородности данных и отсутствия доверительных интервалов в большинстве исследований метаанализ не был проведен.

**Результаты:**

В анализ включили 76 статей из 50 стран. Они охватывали 74 исследования, проведенные в период между 1995 и 2014 годами, включая в общей сложности 202 607 представителей сексуальных и гендерных меньшинств. Качество данных было относительно низким из-за отсутствия стандартизированных критериев и иногда по причине использования небольшой и нерандомизированной выборки. В исследованиях, где все сексуальные и гендерные меньшинства анализировались в виде единой популяции, распространенность физического и сексуального насилия варьировалась от 6% (в исследовании, включающем 240 человек) до 25% (49/196 человек) и от 5,6% (28/504) до 11,4% (55/484) соответственно. Для транссексуалов распространенность варьировалась от 11,8% (около 34 человек) до 68,2% (75/110) и от 7,0% (в исследовании, включающем 255 человек) до 49,1% (54/110).

**Вывод:**

Необходимы дополнительные данные о распространенности, факторах риска и последствиях физического и сексуального насилия, мотивированных сексуальной ориентацией и гендерной индивидуальностью, в разных географических и культурных условиях. Национальная политика и меры по предотвращению насилия должны охватывать сексуальные и гендерные меньшинства.

## Introduction

On 17 June 2011, the United Nations (UN) Human Rights Council passed a resolution that expressed grave concern at violence and discrimination against individuals based on their sexual orientation and gender identity.^1^ This first-ever UN resolution on sexual orientation and gender identity requested a report by the Office of the High Commissioner for Human Rights. It was published in November 2011 and stated:

“Homophobic and transphobic violence has been recorded in all regions. Such violence may be physical (including murder, beatings, kidnappings, rape and sexual assault) or psychological (including threats, coercion and arbitrary deprivations of liberty). These attacks constitute a form of gender-based violence, driven by a desire to punish those seen as defying gender norms.”^2^

An updated 2014 resolution confirmed these conclusions and culminated in the designation of an UN Independent Expert on sexual orientation and gender identity in September 2016.^3^^–^^5^

Although the UN recognized violence against individuals based on their sexual orientation and gender identity as a form of gender-based violence, we do not know whether such violence is characterized by the same gender dynamics and motivations as gender-based violence against women or if it follows a different path.^6^^–^^9^

Violence against individuals based on their sexual orientation is one of the ways in which sexual stigma is expressed.^10^ Sexual stigma based on perceived sexual orientation emerges from a society’s shared belief system in which homosexuality is denigrated and discredited as invalid relative to heterosexuality. Stigma based on gender identity works along the same lines of a gendered society in which only two gender possibilities, masculine or feminine, are perceived as valid. This stigma is incorporated by a society and enacted by its institutions.^10^ In many countries, for example, laws criminalize sexual and gender minorities directly or indirectly on the grounds of morality or promotion of non-traditional values. This can result in physical punishment, death penalty, arbitrary arrest and torture, ill-treatment in health facilities and forced sterilization.^11^^–^^13^ Discriminatory health policies have also resulted in unnecessary gender-conformation operations in intersex babies.^14^ Individuals identified as sexual and gender minorities (Box 1) and may internalize the negative attitudes and values of society. This internalized homophobia or transphobia has detrimental effects on their mental health and might result in self-harm or violence among individuals.^15^^–^^17^

Box 1 **Definitions used in the systematic review of physical and sexual violence motivated by perception of sexual orientation and gender identity**

**Sexual and gender minority**

People identifying themselves as homosexual, bisexual or nonbinary sexual, such as pansexual and polysexual, or people engaging in homosexual, bisexual or nonbinary sexual behaviour or identifying with or expressing as a different gender than the one assigned at birth (male, female or another), or intersex people.

**Sexual orientation**

Refers to each person’s capacity for profound emotional, affectional and sexual attraction to (and intimate and sexual relations with) individuals of any sex.

**Gender identity or gender expression**

Refers to a person’s deeply felt internal and individual experience of gender, which may or may not correspond with the sex assigned at birth. It includes both the personal sense of the body – which may involve, if freely chosen, modification of bodily appearance or function by medical, surgical or other means – as well as other expressions of gender, including dress, speech and mannerisms.

**Homosexual**

A person who has sexual relations with or sexual attraction to people of the same sex.

Gay

The term gay can refer to same-sex sexual attraction, same-sex sexual behaviour and same-sex cultural identity. Unless individuals or groups self-identify as gay, the expression men who have sex with men should be used.

Lesbian

A lesbian is a woman attracted to other women. She may or may not be having sex with women, and a woman having sex with women may or may not be a lesbian. The term women who have sex with women should be used unless individuals or groups self-identify as lesbians.

**Bisexual**

A person who is attracted to or has sexual relations with both men and women.

**Transgender**

People whose gender identity and expression does not conform to the norms and expectations traditionally associated with their sex at birth. It includes individuals who have received gender reassignment surgery, individuals who have received gender-related medical interventions other than surgery (e.g. hormone therapy) and individuals who identify as having no gender, multiple genders or alternative genders.

**Intersex**

An individual with both male and female biological attributes (primary and secondary sexual characteristics).

**Gender non-conforming or gender variant or queer**

A person who challenges (or is not conforming to) prevailing gender norms and expectations or to heterosexual norms.

Note: Except for sexual and gender minority and queer, all definitions were based on the 2015 Joint United Nations Programme on HIV and AIDS terminology guidelines.^23^

Several comprehensive reviews have demonstrated that sexual and gender minorities are more likely to be victims of physical and sexual violence than the general population.^18^^–^^21^ However, these did not report whether the victims perceived the violence being against their sexual orientation and gender identity. Our study aimed to review the research evidence on the prevalence of physical and sexual violence motivated by perception of sexual orientation, gender identity or gender expression among sexual and gender minorities. We distinguished this from violence inflicted on a random member of the general population or violence experienced by sexual and gender minorities, but not specifically perceived to be motivated by their sexual orientation or gender identity.

## Methods

Our review followed the Preferred Reporting Items for Systematic Reviews and Meta-Analyses guidelines.^22^ The protocol for this review has not been registered on the PROSPERO register of systematic reviews, but is available on request.

We searched nine bibliographic databases (PubMed®, Embase®, Web of Science, Africa Wide Information, CINAHL, LILACS, Popline, Sociological Abstracts and GenderWatch) for articles published from 1 January 2000 to 28 April 2016. We used a combination of medical subject headings and text words (Box 2), with no language restrictions. These searches were supplemented by a scan of the citations in the articles for studies not found in the search and by consultation with individual experts about their knowledge of other studies.

Box 2 **PubMed® search strategy used in the systematic review of physical and sexual violence motivated by perception by sexual orientation and gender identity**

1# homosexuality[Mesh] OR bisexuality[Mesh] OR transsexualism[Mesh] OR “transgendered persons”[Mesh] OR homophobia[Mesh] OR “Health Services for Transgendered Persons”[Mesh] OR “Disorders of Sex Development”[Mesh] OR “gender identity”[Mesh] OR homosexuality[TW] OR homosexual[TW] OR homosexual*[TW] OR “homo-sexual”[TW] OR homo-sexual*[TW] OR (“same sex”[TW] NOT twins) OR (“same sex” AND twins AND homosexuality) OR “non heterosexual”[TW] OR “same gender loving”[TW] OR “same sex attracted”[TW] OR queer*[TW] OR LBGT[TW] OR LBGT*[TW] OR LGBT[TW] OR LGBT*[TW] OR GLBT*[TW] OR GLB*[TW] OR LGB*[TW] OR LGBTQ*[TW] OR LGBTI*[TW] OR sexual orientation and gender identity[TW] OR sexual minorit*[TW] OR gender minorit*[TW] OR “sexual orientation”[TW] OR “gender identity”[TW] OR gay[TW] OR gays[TW] OR (“MSM”[TW] NOT “metal-semiconductor-metal”) OR “men who have sex with men”[TW] OR (“MSW”[TW] NOT waste) OR “male sex workers”[TW] OR sissy[TW] OR sissies[TW] OR “money boys”[TW] OR “kwandengue”[TW] OR “male street laborers”[TW] OR “mashoge”[TW] OR lesbian[TW] OR lesbian*[TW] OR lesbians*[TW] OR “WSW”[TW] OR “women who have sex with women”[TW] OR tomboy*[TW] OR “pengkids”[TW] OR bisexuality[TW] OR bisexual*[TW] OR bi-sexual*[TW] OR transgender*[TW] OR trans-gender*[TW] OR transvestism[TW] OR transvestite[TW] OR transsexual*[TW] OR transsexualism*[TW] OR “trans man”[TW] OR “trans men”[TW] OR “trans women”[TW] OR “trans woman”[TW] OR “transman”[TW] OR “transmen”[TW] OR “transwomen”[TW] OR “transwoman”[TW] OR transgendered[TW] OR “sex change” [TW] OR “sex reassignment surgery”[TW] OR “gender adjustment surgery”[TW] OR cross-dress*[TW] OR “gender variant”[TW] OR “gender atypical”[TW] OR “gender identity disorder”[TW] OR transgenderist[TW] OR “drag queens”[TW] OR “drag kings”[TW] OR “gender queer”[TW] OR “gender-queer”[TW] OR “gender dysphoria”[TW] OR “hijra”[TW] OR “aravanis” [TW] OR “kothi”[TW] OR “Kathoy”[TW] OR “Kathoey”[TW] OR “fa’afafine”[TW] OR “sworn virgins”[TW] OR “two-spirit”[TW] OR “Metis”[TW] OR “mak nyah”[TW] OR “travesty”[TW] OR “koti”[TW] OR “mahuvahine”[TW] OR “mahu”[TW] OR “waria”[TW] OR “bantut”[TW] OR “nadleehi”[TW] OR “berdache”[TW] OR “xanith”[TW] OR (intersex AND human) OR (intersex* AND human) OR bigender[TW] OR pansexual[TW] OR omnisexual[TW] OR “questioning people”[TW] OR “questioning youth”[TW] OR homophob*[TW] OR homo-phob*[TW] OR transphob*[TW] OR trans-phob*[TW] OR “anti homosexual bias”[TW] OR “anti gay bias”[TW]

2# violence[MeSH] OR “sex offenses”[MeSH] OR homicide[MeSH] OR rape[MeSH] OR aggression[MeSH] OR “crime victims”[MeSH] OR Stalking[MeSH] OR “battered women”[MeSH] OR “spouse abuse”[MeSH] OR violence[TW] OR violen*[TW] OR rape[TW] OR IPV[TW] OR SGBV[TW] OR assault*[TW] OR victimi*ation[TW] OR revictimi*ation[TW] OR re-victim*ation[TW] OR stalking[TW] OR “hate crimes”[TW] OR “hate crime”[TW] OR “relationship abuse”[TW] OR “dating abuse”[TW] OR “partner abuse”[TW] OR “physical abuse”[TW] OR “psychological abuse”[TW]

3# 1# AND 2#

Note: Search strategies for other databases used (Embase®, Web of Science, Africa Wide Information, CINAHL, LILACS, Popline, Sociological Abstracts and GenderWatch) are available from the corresponding author.

Studies were eligible for inclusion if they included people belonging to a sexual or gender minority. We included both peer-reviewed and grey literature reporting studies that measured the prevalence of physical and sexual violence perceived as being motivated by sexual orientation, gender identity or gender expression. We excluded intimate partner violence and self-harm. Studies had to be published from 2000 to the search date, refer to data collected after 1995 and include at least 50 participants.

Two researchers screened the identified abstracts. When there was doubt or disagreement about whether an article met the inclusion criteria, the article was taken to the next stage of screening. The researchers then independently assessed the full text of potentially eligible studies. If needed, we contacted the authors of the articles for further information.

After initial screening, we appraised the included studies for quality. The criteria were: sampling method, sample representativeness, description of the population, completeness of the data, description of the methods, reliability of the data, and controls for confounding. We categorized studies as high quality if six to seven criteria were adequate, medium quality if three or five criteria were adequate and low quality if none to two criteria were adequate. None of the studies were excluded based on this quality assessment. We minimized publication bias across studies by including grey literature and consulting with experts.

Two researchers independently extracted details of the studies into a database. The data collected were: country and area; data collection period; study type and sampling method; description of study population; terminology of violence used to elicit responses from participants; time periods of experiences of violence (ever in lifetime, specific dates or time periods); participants’ perceptions of motivation for violence; sample size; and number and percentage of respondents affected by different types of violence. The outcome of interest for the review was the prevalence of physical and sexual violence motivated by perception of sexual orientation and gender identity. However, such violence was not the primary outcome in most of the studies.

We made a descriptive summary of the prevalence data in tables and charts. Although the UN resolution^2^ included sexual violence within physical violence, most studies reported them separately. Where possible and relevant, we conducted separate descriptive analyses of subgroups of sexual and gender minorities. The results of the studies were highly heterogeneous, due to variability in the sampling (definition of the population and sampling methods) and the descriptions of violence used to gather data from participants. In view of this heterogeneity and the absence of confidence intervals in most studies (reported in only six), we did not attempt a meta-analysis.

## Results

### Study selection

**Fig. 1:**
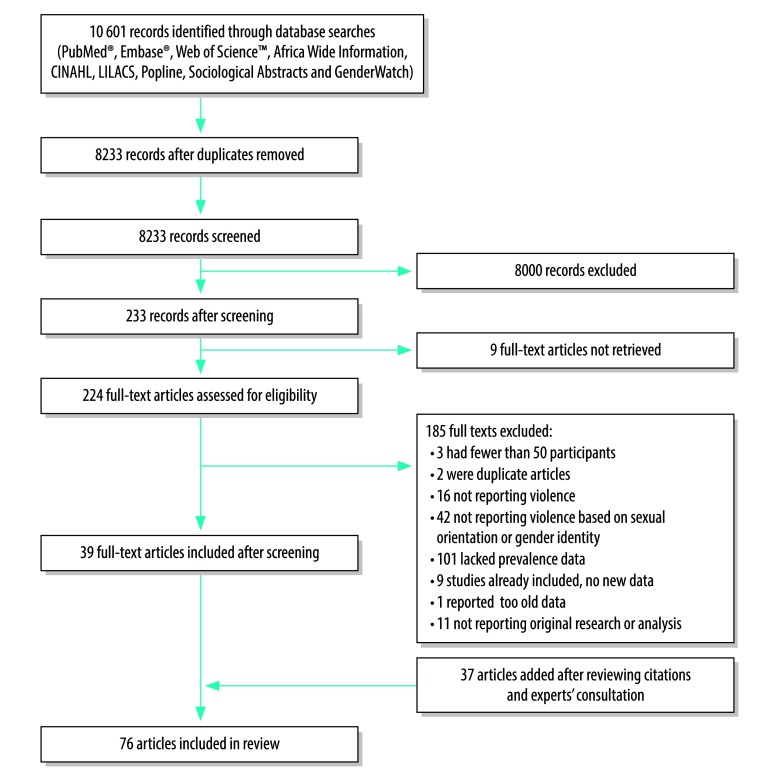
Flowchart for selection of articles in the systematic review of physical and sexual violence motivated by perception of sexual orientation and gender identity

Our literature search yielded 10 601 references, of which 8233 were unique entries. Next, we excluded 8000 articles after screening titles and abstracts. Of the 233 references that potentially met the inclusion criteria, nine could not be retrieved, and of the 224 retrieved texts, 185 were excluded for different reasons (Fig. 1). We added 37 articles and reports after citation tracking and consulting with experts. In total 76 articles were included in the review.^24^^–^^100^ Seven articles were categorized as low quality, 55 as medium and 14 as high quality (Table 1). 

**Table 1:** Quality appraisal of the 76 articles (74 studies) included in the systematic review of physical and sexual violence motivated by perception of sexual orientation and gender identity

Publication	Sampling method(s)	Sample representativeness	Description of population	Follow up or completeness of data	Description of methods	Reliability of data	Controlled for confounding	Score
D'Augelli et al., 2001^42^	N	Y	Y	Y	Y	Y	Y	High
Diaz et al., 2001^48^	Y	Y	Y	Y	Y	Y	Y	High
Lombardi et al., 2001^78^	N	Y	Y	N	Y	Y	Y	Medium
D'Augelli et al., 2002^43^	N	Y	Y	Y	Y	Y	Y	High
Kosciw, 2002^64^	N	Y	Y	N	Y	Y	Y	Medium
Carrara et al., 2003^34^	N	N	Y	N	Y	Y	N	Medium
Jarman et al., 2003^62^	N	Y	Y	N	Y	Y	N	Medium
Morris et al., 2003^81^	N	Y	Y	N	Y	Y	N	Medium
Rose, 2003^94^	N	N	Y	N	N	Y	N	Low
Huebner et al., 2004^59^	N	Y	Y	Y	Y	N	Y	Medium
Kosciw 2004^65^	N	Y	Y	N	Y	Y	Y	Medium
Carrara et al., 2005^35^	N	N	Y	N	Y	Y	N	Medium
Fígari et al., 2005^50^	N	N	Y	Y	Y	Y	N	Medium
Hillier et al., 2005^57^	N	Y	Y	Y	Y	Y	N	Medium
Carrara et al., 2006^36^	N	N	Y	N	Y	Y	N	Medium
Clements-Nolle et al., 2006^40^	N	Y	Y	Y	Y	Y	Y	High
D'Augelli et al., 2006^44^	N	Y	Y	N	Y	Y	N	Medium
Jones et al., 2006^63^	N	N	Y	N	Y	Y	N	Medium
Kosciw et al., 2006^66^	N	Y	Y	N	Y	Y	Y	Medium
Ortiz-Hernandez et al., 2006^88^	N	Y	Y	N	Y	Y	Y	Medium
Pitts et al., 2006^91^	N	Y	Y	N	Y	N	N	Medium
van San et al., 2006^100^	N	N	Y	Y	Y	Y	N	Medium
Carrara et al., 2007^37^	N	N	Y	N	Y	Y	N	Medium
Couch et al., 2007^41^	N	Y	Y	Y	Y	N	N	Medium
Lippl, 2007^76^	N	Y	Y	Y	Y	Y	N	Medium
Poelman et al., 2007^92^	N	Y	Y	N	Y	Y	N	Medium
Barrientos et al., 2008^25^	N	N	Y	N	Y	Y	N	Medium
Cadiou et al., 2008^33^	N	Y	Y	Y	Y	Y	Y	High
Kosciw et al., 2008^67^	N	Y	Y	N	Y	Y	Y	Medium
Lampinen et al., 2008^72^	N	Y	Y	N	Y	Y	Y	Medium
Paterson et al., 2008^89^	N	Y	Y	Y	Y	N	N	Medium
Scottish Transgender Alliance, 2008^95^	N	Y	Y	N	N	N	N	Low
Brigeiro et al., 2009^30^	N	N	Y	Y	Y	Y	N	Medium
Greytak, 2009^52^	N	Y	Y	N	Y	Y	N	Medium
Herek, 2009^54^	Y	Y	Y	Y	Y	Y	Y	High
Lippl, 2009^77^	N	Y	Y	Y	Y	Y	N	Medium
Hillier et al., 2010^58^	N	Y	Y	Y	Y	N	Y	Medium
Kosciw et al., 2010^68^	N	Y	Y	N	Y	Y	Y	Medium
Nuttbrock et al., 2010^84^	N	Y	Y	N	Y	Y	Y	Medium
Chapman et al., 2011^39^	N	N	Y	N	Y	N	N	Low
Hightow-Weidman et al., 2011^56^	N	Y	Y	N	Y	Y	Y	Medium
Nemoto et al., 2011^83^	N	Y	Y	N	Y	Y	N	Medium
Barrientos et al., 2012^26^	N	N	Y	N	Y	Y	N	Medium
Brito et al., 2012^31^	N	N	Y	N	Y	Y	N	Medium
Guasp, 2012^53^	N	Y	Y	N	N	N	N	Low
Iosa et al., 2012^60^	N	N	Y	N	Y	Y	N	Medium
Kosciw et al., 2012^69^	N	Y	Y	N	Y	Y	Y	Medium
Leonard et al., 2012^74^	N	Y	Y	N	Y	N	N	Medium
Levitt et al., 2012^75^	N	Y	Y	N	Y	N	N	Medium
McNeil et al., 2012^79^	N	Y	Y	Y	Y	Y	N	Medium
Motmans et al., 2012^82^	N	Y	Y	N	N	N	N	Low
Oogachaga, 2012^86^	N	N	N	Y	N	Y	N	Low
Testa et al., 2012^99^	N	Y	Y	N	Y	Y	Y	Medium
Chamberland et al., 2013^38^	N	Y	N	Y	Y	Y	N	Medium
de Sousa et al., 2013^47^	Y	N	Y	N	Y	N	N	Medium
Pelullo et al., 2013^90^	N	N	Y	Y	Y	N	Y	Medium
Aho et al., 2014^24^	Y	Y	Y	Y	Y	Y	Y	High
Boza et al., 2014^29^	N	Y	Y	Y	Y	Y	Y	High
de Deus 2014^46^	Y	Y	Y	N	Y	Y	N	Medium
Herrick et al., 2014^55^	Y	Y	Y	Y	Y	Y	Y	High
Ivanković et al., 2014^61^	N	Y	Y	Y	Y	Y	Y	High
Kosciw et al., 2014^70^	N	Y	Y	N	Y	Y	Y	Medium
Lea et al., 2014^73^	N	Y	Y	Y	Y	Y	Y	High
Mereish et al., 2014^80^	N	N	Y	Y	Y	Y	N	Medium
Nuttbrock et al., 2014^85^	N	Y	Y	Y	Y	Y	Y	High
Scruton, 2014^96^	N	Y	Y	Y	Y	N	N	Medium
Smith et al., 2014^97^	N	Y	Y	Y	Y	Y	N	Medium
Strizzi et al., 2014^98^	N	Y	Y	N	Y	Y	N	Medium
Bauer et al., 2015^28^	Y	Y	Y	Y	Y	Y	Y	High
Burks et al., 2015^32^	N	N	Y	N	Y	Y	Y	Medium
Ferlatte et al., 2015^49^	N	Y	Y	N	Y	Y	Y	Medium
Goldbach et al., 2015^51^	N	Y	Y	N	Y	Y	Y	Medium
Barrientos et al., 2016^27^	N	Y	Y	Y	Y	Y	N	Medium
D’haese et al., 2016^45^	N	Y	Y	Y	Y	Y	Y	High
Kramer et al., 2016^71^	N	Y	Y	N	Y	Y	Y	Medium
Rodriguez-Madera et al., 2016^93^	Y	N	Y	N	N	N	N	Low

Notes: Y indicates that the study met the criterion adequately; N that the study did not. We categorized studies as high quality if six to seven criteria were adequate, medium quality if three or five criteria were adequate and low quality if none to two criteria were adequate.

### Study characteristics

Of the 76 articles, 56 were in English language, seven in Spanish, six in Portuguese, three in Dutch, two in French and two in German. Data were from 50 countries: United States of America (USA; 27 articles), Australia (7 articles), Brazil (6 articles), Canada (5 articles), United Kingdom of Great Britain and Northern Ireland (5 articles), Argentina (3 articles), Belgium (3 articles), Chile (3 articles), Mexico (2 articles), Germany (2 articles), USA and Canada (2 articles); Australia and New Zealand (1 article), Spain and USA (1 article); 38 European countries (1 article); and Colombia, Côte d’Ivoire, Croatia, France, Italy, Netherlands, Rwanda and Singapore (1 article each).

Thirty-six publications were peer-reviewed articles, 38 were study reports, one was a dissertation and one a book chapter.

The 76 articles were based on 74 studies conducted between 1995 and 2014, including a total of 202 607 sexual and gender minorities participants. Sixty-three studies used a convenience sample, four used respondent-driven sampling, four used venue-based or time-location sampling, one random digit dialling and two used mixed methods (Table 2; available at: http://www.who.int/bulletin/volumes/96/1/17-197251). 

**Table 2:** Main characteristics of the 76 articles (74 studies) included in the systematic review of physical and sexual violence motivated by perception of sexual orientation and gender identity

Author and year	Area, country	Data-collection period	Study population^a^	Study type; sampling method	Type of violence^b^	Sample, no.	No. (%) affected by violence^c^
D'Augelli et al., 2001^42^	USA and Canada	1997–1998	Lesbian gay and bisexual people (≥ 60 years old)	Convenience; cross-sectional	Lifetime experience of physical victimization (object being thrown), physical assault (punched, kicked, or beaten), or sexual assault or rape	All groups: 416	Object thrown: 46 (11.2%) Punched, kicked, beaten: 62 (15.6%) Sexual assault or rape: 29 (7.3%)
						Male: 297	Object thrown: 34 (12.0%) Punched, kicked, beaten: 58 (21.6%) Sexual assault or rape: 27 (9.4%)
						Female: 119	Object thrown: 10 (9.0%) Punched, kicked, beaten: 4 (3.6%) Sexual assault or rape: 2 (1.8%)
Diaz et al., 2001^48^	New York, Los Angeles and Miami, USA	1998–1999	Gay and bisexual people (Latino)	Venue-based; cross-sectional	Ever experience in childhood and adulthood of physical assault	912	Physical assault in childhood: 18% (95% CI: 15–21); in adulthood: 10% (95% CI: 7–12)
Lombardi et al., 2001^78^	USA	1996 −1997	Transgender people	Convenience; cross-sectional	In the past 30 days, 12 months or ever experienced assault with a weapon, assault without a weapon, rape or attempted assault	402	Assault without weapon in past 30 days: 7 (1.7%); past 12 months: 26 (6.5%); lifetime: 78 (19.4%) Assault with weapon in past 30 days: 5 (1.2%); past 12 months: 12 (3.0%); lifetime: 41 (10.2%) Object thrown in past 30 days: 9 (2.2%); past 12 months: 26 (6.5%); lifetime: 70 (17.4%) (Attempted) rape in past 30 days: 2 (0.5%); past 12 months: 11 (2.7%); lifetime: 55 (13.7%)
D'Augelli et al., 2002^43^	USA, Canada and New-Zealand	1995‒1997	Lesbian, gay and bisexual people (age ≤ 21 years)	Convenience; cross-sectional	Lifetime experience of physical victimization: (object being thrown, punched, kicked, or beaten) or sexual assault	All groups: 350	Object thrown: 35/299 (11.7%) Punched, kicked, beaten: 32/301 (10.7%) Sexual assault: 14/292 (4.8%)
						Male: 193	Object thrown: 24/165 (14.5%) Punched, kicked, beaten: 24/165 (14.5%) Sexual assault: 9/159 (5.7%)
						Female: 154	Object thrown: 10/134 (7.5%) Punched, kicked, beaten: 9/136 (6.6%) Sexual assault: 5/133 (4.0%)
Kosciw, 2002^64^	USA	2001	Lesbian, gay, bisexual and transgender youth (13–20 years old)	Convenience; cross-sectional	In the past school year, been physically assaulted at school	All groups: 904	Physical assault based on sexual orientation: N/A (21.1%); gender expression N/A (13.7%)
						Male: 458	Physical assault based on sexual orientation: N/A (23.6%); gender expression: N/A (14.2%)
						Female: 385	Physical assault based on sexual orientation: N/A (15.8%); gender expression: N/A (10.5%)
						Transgender: 28	Physical assault based on sexual orientation: N/A (31.6%); gender expression: N/A (35.1%)
Carrara et al., 2003^34^	Rio de Janeiro, Brazil	2003	Lesbian, gay, bisexual and transgender people	Convenience; cross-sectional	Lifetime experience of physical aggression or sexual violence	All groups: 403	Physical aggression: 67 (16.6%) Sexual violence: 24 (6.0%)
						Gay: 215	Physical aggression: 42 (19.5%) Sexual violence: 17 (7.6%)
						Lesbian: 102	Physical aggression: 10 (9.8%) Sexual violence: 1 (1.0%)
						Bisexual: 41	Physical aggression: 3 (7.3%) Sexual violence: 1 (2.4%)
						Transgender: 26	Physical aggression: 11 (42.3%) Sexual violence: 3 (11.5%)
Jarman et al., 2003^62^	Northern Ireland	2002–2003	Lesbian, gay and bisexual people	Convenience; cross-sectional	Lifetime and in the past 2 years experience of having object thrown, physical or sexual assault	186	Object thrown in past 2 years: 45 (24.2%); lifetime: 65 (35.0%) Physical assault in past 2 years: 46 (24.7%); lifetime: 56 (30.1%) Sexual assault or rape in past 2 years: 10 (5.4%); lifetime: 18 (9.7%)
Morris et al., 2003^81^	USA	1994–1995	Lesbian and bisexual women	Convenience; cross-sectional	Lifetime experience of physically attack, sexual assault or rape	2431	Physical attack: N/A (6.5%) Sexual assault or rape: N/A (˂ 2.0%)
Rose, 2003^94^	Saint-Louis, USA	N/A	Lesbian people	Convenience; cross-sectional	In the past 12 months been assaulted with a weapon, or experienced physical or sexual assault	229	Sexual assault: N/A (7.4%) Physical assault: N/A (5.2%) Assault with a weapon: N/A (1.7%)
Huebner et al., 2004^59^	Phoenix, Albuquerque, New Mexico and Austin, USA	1996–1997	Gay and bisexual people	Convenience; cross-sectional	In the past 6 months experienced physical violence	1210	Physical violence: 58 (4.8%; 95% CI: 3.6–6.0)
Kosciw 2004^65^	USA	2003	Lesbian, gay, bisexual and transgender youth (13–20 years old)	Convenience; cross-sectional	In the past school year, been physically assaulted at school	887	Physical assault based on sexual orientation: N/A (17.0%); gender expression: N/A (11.5%)
Carrara et al., 2005^35^	Rio de Janeiro, Brazil	2004	Lesbian, gay, bisexual and transgender people	Convenience; cross-sectional	Lifetime experience of physical aggression or sexual violence	All groups: 504	Physical aggression: 94 (18.7%) Sexual violence: 28 (5.6%)
Fígari et al., 2005^50^	Buenos Aires, Argentina	2004	Lesbian, gay, bisexual and transgender people	Convenience; cross-sectional	Lifetime experience of physical aggression or sexual violence	All groups: 484	Physical aggression: 92 (19.0%) Sexual violence: 55 (11.4%)
						Gay: 279	Physical aggression: 53 (19.0%) Sexual violence: 29 (10.4%)
						Lesbian: 106	Physical aggression: 17 (16.0%) Sexual violence: 14 (13.2%)
						Bisexual: 63	Physical aggression: 3 (4.8%) Sexual violence: 5 (7.9%)
						Transgender: 32	Physical aggression: 18 (56.3%) Sexual violence: 7 (21.9%)
Hillier et al., 2005^57^	Australia	2003–2004	Same sex attracted people (14–21 years old)	Convenience; cross-sectional	Lifetime experience of physical abuse	All groups: 1749	Physical abuse: N/A (15%)
						Male: 1106	Physical abuse: N/A (19%)
						Female: 643	Physical abuse: N/A (9%)
Carrara et al., 2006^36^	São Paulo, Brazil	2005	Lesbian, gay, bisexual and transgender people	Convenience; cross-sectional	Lifetime experience of physical aggression or sexual violence	All groups: 721	Physical violence: 133 (18.4%) Sexual violence: 46 (6.4%)
						Homosexual and bisexual male: 413	Physical violence: 102/411 (24.8%) Sexual violence: 24 (5.9%)
						Homosexual and bisexual female: 219	Physical violence: 11 (4.9%) Sexual violence: 12 (5.6%)
						Transgender: 80	Physical violence: 43 (53.8%) Sexual: 19 (23.8%)
Clements-Nolle et al., 2006^40^	San Francisco, USA	1997	Transgender people	Targeted, respondent driven and convenience; cross-sectional	Lifetime experience of physical abuse or beating	511	Physical violence: 184 (35.7%)
D'Augelli et al., 2006^44^	New York, USA	N/A	Lesbian, gay and bisexual people (15–19 years old)	Convenience; longitudinal	Lifetime experience of physical violence (punched, kicked, or beaten or hurt with a knife, gun, bat, or some other weapon) or sexual violence (sexual abuse or rape)	Male: 274	Physical violence: N/A (15%) Sexual violence: N/A (14%)
						Female: 254	Physical violence: N/A (7%) Sexual violence: N/A (5%)
Jones et al., 2006^63^	Argentina, Buenos Aires	2005	Lesbian, gay, bisexual and transgender people	Convenience; cross-sectional	Lifetime experience of physical aggression or sexual violence	Gay: 289	Physical aggression: 39 (13.5%) Sexual violence: 11 (3.8%)
						Lesbian: 138	Physical aggression: 14 (10.1%) Sexual violence: 11 (8.0%)
						Bisexual: 90	Physical aggression: 12 (13.5%) Sexual violence: 5 (5.6%)
						Transgender: 67	Physical aggression: 35 (52.2%) Sexual violence: 23 (34.3%)
Kosciw et al., 2006^66^	USA	2005	Lesbian, gay, bisexual and transgender youth (13–20 years old)	Convenience; cross-sectional	In the past 12 months, been physical assaulted at school	1732	Physical assault based on sexual orientation: 302/1717 (17.6%); gender expression: 201/1706 (11.8%)
Ortiz-Hernandez et al., 2006^88^	Mexico City, Mexico	2001	Lesbian, gay and bisexual people	Convenience; cross-sectional	(i) Been hit or beaten in childhood and adolescence due to gender stereotypes transgression (ii) Ever or in the past 12 months experienced physical and sexual violence in adulthood (age > 18 years)	*In childhood *	
						All groups: 506	Hit or beaten from age 6–11 years: N/A (8%); age 12–17 years: N/A (6%)
						Male: 318	Hit or beaten from age 6–11 years: N/A (11%); age 12–17 years: N/A (7%)
						Female: 188	Hit or beaten from age 6–11 years: N/A (2%); age 12–17 years: N/A (4%)
						*In adulthood*	
						All groups: 494 (past 12 months); 422 (lifetime)	Object thrown in past 12 months: N/A (8%); lifetime: N/A (15%) Physical aggression: in past 12 months: N/A (7%); lifetime: N/A (16%) Physical injury with a weapon in past 12 months: N/A (3%); lifetime: N/A (6%) Rape: in past 12 months: N/A (3%); lifetime: N/A (9%)
						Male: 312 (past 12 months); 264 (lifetime)	Object thrown in past 12 months: N/A (7%); lifetime: N/A (18%) Physical aggression in past 12 months: N/A (5%); lifetime: N/A (17%) Physical injury with a weapon in past 12 months: N/A (2%); lifetime: N/A (6%) Rape in past 12 months: N/A (4%); lifetime: N/A (10%)
						Female: 182 (past 12 months); 158 (lifetime)	Object thrown in past 12 months: N/A (8%); lifetime: N/A (10%) Physical aggression in past 12 months: N/A (10%); lifetime: N/A (14%) Physical injury with a weapon in past 12 months: N/A (4%); lifetime: N/A (5%) Rape in past 12 months: N/A (3%); lifetime: N/A (8%)
Pitts et al., 2006^91^	Australia	2005	Sexual and gender minorities	Convenience; cross-sectional	Lifetime experience of physical attack or other kind of violence, object thrown, rape or sexual assault	Male: 3429	Physical violence: N/A (17.3%) Object thrown: N/A (14.0%) Rape: N/A (4.1%) Sexual assault: N/A (3.7%)
						Female: 1929	Physical violence: N/A (7.2%) Object thrown: N/A (7.9%) Sexual assault: N/A (2.7%) Rape: N/A (2.5%)
						Female-to-male transgender people: 34	Physical violence: N/A (11.8%) Object thrown: N/A (14.7%) Rape: N/A (8.8%) Sexual assault: N/A (8.8%)
						Male-to-female transgender people: 66	Physical violence: N/A (18.2%) Object thrown: N/A (12.1%) Rape: N/A (3.0%) Sexual assault: N/A (10.6%)
						Intersex male: 11	Physical violence: N/A (18.2%) Object thrown: N/A (27.3%) Rape: N/A (18.2%) Sexual assault: N/A (18.2%)
						Intersex female: 7	Physical violence: N/A (28.6%) Object thrown: N/A (28.6%) Rape: 0 (0%) Sexual assault: N/A (28.6%)
van San et al., 2006^100^	Netherlands	N/A	Homosexual males and females	Convenience; cross-sectional	Lifetime experience of physical violence	761	Physical violence: 24 (3.3%)
Carrara et al., 2007^37^	Recife, Brazil	2006	Lesbian, gay, bisexual and transgender people	Convenience; cross-sectional	Lifetime experience of physical aggression or sexual violence	All groups 544:	Physical violence: 113 (20.8%) Sexual violence: 55 (10.2%)
						Homosexual male: 269	Physical aggression: 65 (24.2%) Sexual violence: 32 (12.1%)
						Bisexual male: 53	Physical aggression: 12 (22.6%) Sexual violence: 3 (5.8%)
						Homosexual female: 113	Physical aggression: 9 (8.6%) Sexual violence: 4 (3.8%)
						Bisexual female: 49	Physical aggression: 30 (6.1%) Sexual violence: 30 (6.1%)
						Transgender: 36	Physical aggression: 20 (57.1%) Sexual violence: 11 (30.6%)
Couch et al., 2007^41^	Australia and New Zealand	2006–2007	Transgender people	Convenience; cross-sectional	Lifetime experience of physical attack or other kind of violence, object being thrown, sexual assault or rape	253	Physical attack: 47 (18.6%) Object thrown: 37 (14.6%) Sexual assault: 29 (11.5%) Rape: 25 (9.9%)
Lippl, 2007^76^	Germany	2007–2008	Homosexual and bisexual men	Convenience; cross-sectional	In the past 12 months been physically assaulted	23 949	Physical injury: N/A (8.6%)
Poelman et al., 2007^92^	Brussels, Belgium	2006	Lesbian, gay and bisexual people	Convenience; cross-sectional	Lifetime experience of physical aggression, sexual assault or rape	377	Physical aggression: 34 (9.0%) Sexual assault or rape: 8 (2.1%)
Barrientos et al., 2008^25^	Santiago, Chile	2007	Lesbian, gay, bisexual and transgender people	Cross-sectional; convenience	Lifetime experience of physical aggression or sexual violence	All groups: 400	Physical aggression: 91 (22.8%) Sexual violence: 43 (10.8%)
						Lesbian: 133	Physical aggression: 23 (17.3%) Sexual violence: 12 (9.0%)
						Gay: 193	Physical aggression: 51 (26.4%) Sexual violence: 18 (9.3%)
						Bisexual: 55	Physical aggression: 8 (14.5%) Sexual violence: 5 (9.1%)
						Transgender: 19	Physical aggression: 9 (47.4%) Sexual violence: 8 (42.1%)
Cadiou et al., 2008^33^	France	2003–2004	Lesbian and gay women	Convenience; cross-sectional	Lifetime experience of physical violence or rape in different contexts	1740	Physical violence from family: 30 (1.67%); friends: 11 (0.61%); neighbours: 39 (2.18%); in public life: 92 (5.13%); by government services: 18 (1.00%); at workplace: 2 (0.11%); by police: 4 (0.22%). Rape by family 17 (0.95%); friends: 4 (0.22%); neighbours: 4 (0.22%); in public life: 6 (0.33%); at workplace: 4 (0.22%)
Kosciw et al., 2008^67^	USA	2007	Lesbian, gay, bisexual and transgender youth (13–21 years old)	Convenience; cross-sectional	In the past 12 months, been physically assaulted at school	6209	Physical assault based on sexual orientation: N/A (22.1%) gender expression: N/A (14.2%)
Lampinen et al., 2008^72^	Vancouver, Canada	1995–2004	Men who have sex with men (15–30 years old, HIV-negative)	Convenience; longitudinal	Ever or in the past 12 months experienced physical abuse	521	Physical abuse in past 12 months: 18 (3,5%); lifetime: 84 (16,1%)
Paterson et al., 2008^89^	United Kingdom of Great Britain and Northern Ireland	N/A	Lesbian, bisexual and transgender women	Convenience; cross-sectional	Ever or in the past 12 months experienced physical violence, grievous bodily harm, attempted murder, rape or other sexual violence	1112	Physical violence or assault in past 12 months: N/A (4.6%); lifetime: N/A (17.9%) Grievous bodily harm in past 12 months: N/A (1.4%); lifetime: N/A (8.3%) Rape in past 12 months: N/A (0.4%); lifetime: N/A (6.0%) Other sexual violence: in past 12 months: N/A (0.7%); lifetime: N/A (7.1%) Attempted murder in past 12 months: N/A (0.4%); lifetime: N/A (4.8%)
Scottish Transgender Alliance, 2008^95^	Scotland	2007	Transgender people	Convenience; cross-sectional	Lifetime experience of physical or sexual abuse in domestic relationships or by a stranger	71	Physical abuse in the home: 8 (11.3%); by a stranger: 12 (16.9%) Sexual abuse in the home: 4 (5.6%); by a stranger: 3 (4.2%)
Brigeiro et al., 2009^30^	Bogotà, Colombia	2007	Lesbian, gay, bisexual and transgender people	Convenience; cross-sectional	Lifetime experience of physical or sexual aggression	Lesbian: 167	Physical aggression: 42 (25.1%) Sexual violence: 20 (12.0%)
						Gay: 419	Physical aggression: 133 (31.7%) Sexual violence: 69 (16.5%)
						Bisexual: 95	Physical aggression: 24 (25.3%) Sexual violence: 14 (14.7%)
						Transgender: 88	Physical aggression: 43 (48.9%) Sexual violence: 29 (33.0%)
Greytak, 2009^52^	USA	2006–2007	Transgender students	Convenience; cross-sectional	In the past year, been physically assaulted in school (punched, kicked, or injured with a weapon)	295	Physical assault based on sexual orientation: N/A (28%); gender expression: N/A (26%)
Herek, 2009^54^	USA	2005	Lesbian, gay and bisexual people	Random digit dialling; cross-sectional	Lifetime experience of violent crime (hit, beaten, physically attacked, sexually assaulted)	All groups: 662	Physical violence: N/A (13.1%; 95% CI: 9.7–17.6) Object thrown: N/A (12.5%; 95% CI: 9.4–16.6)
						Gay: 241	Physical violence: N/A (24.9%; 95% CI: 17.3–34.5) Object thrown: N/A (21.1%; 95% CI: 14.4–29.8)
						Lesbian: 152	Physical violence: N/A (7.1%; 95% CI: 3.7–13.1) Object thrown: N/A (14.6%; 95% CI: 8.9–23.0)
						Bisexual male: 110	Physical violence: N/A (6.9%; 95% CI: 3.1–14.5) Object thrown: N/A (5.6%; 95% CI: 2.4–12.5)
						Bisexual female: 159	Physical violence: N/A (6.7% (95% CI: 3.3–13.0) Object thrown: N/A (6.8%; 95% CI: 3.6–12.5)
Lippl, 2009^77^	Germany	2006–2007	Homosexual and bisexual men	Convenience; cross-sectional	In the past 12 months been physically assaulted	17 477	Physical assault: N/A (4.6%)
Hillier et al., 2010^58^	Australia	2009–2010	Same sex attracted and gender questioning people (14–21 years old)	Convenience; cross-sectional	Lifetime experience of physical abuse	All groups: 3134	Physical abuse: N/A (18%)
						Male: 1265	Physical abuse: N/A (23%)
						Female: 1766	Physical abuse: N/A (14%)
						Gender-questioning: 103	Physical abuse: N/A (31%)
Kosciw et al., 2010^68^	USA	2009	Lesbian, gay, bisexual and transgender youth (13–21 years old)	Convenience; cross-sectional	In the past 12 months been physical assaulted at school	7261	Physical assault based on sexual orientation: N/A (18.8%); gender expression: N/A (12.5%)
Nuttbrock et al., 2010^84^	New York, USA	2004–2009	Male-to-female transgender people	Convenience; longitudinal	Lifetime experience of physical abuse	All ages: 571	Physical violence: 286 (50.1%)
						Age 19–39 years: 333	Physical violence: 171 (51.3%)
						Age 40–59 years: 238	Physical violence: 113 (47.4%)
Chapman et al., 2011^39^	Kigali, Rwanda	2008–2009	Men who have sex with men	Snowball; cross-sectional	Lifetime experience of physical mistreatment	98	Physical violence: 12 (12.2%)
Hightow-Weidman et al., 2011^56^	8 cities, USA	2006–2009	Men who have sex with men (13–24 years old; HIV-positive; non-white)	Convenience; cross-sectional	Lifetime experience of physical violence (hit or beaten up)	351	Physical violence: 57 (16.2%)
Nemoto et al., 2011^83^	San Francisco, USA	2000–2001 2004–2006	Male-to-female transgender sex- workers	Convenience; cross-sectional	Sometimes or almost daily experiences of physical violence	Age 12–18 years: 561	Physical violence sometimes: N/A (39.0%); almost daily: N/A (6.8%)
						Age > 18 years: 561	Physical violence sometimes: N/A (25.0%); almost daily: N/A (0.7%)
Barrientos et al., 2012^26^	Santiago, Chile	2011	Lesbian, gay, bisexual and transgender people	Cross-sectional; convenience	Lifetime experience of physical or sexual aggression	All groups: 196	Physical aggression: 49 (25.0%) Sexual violence: 20 (10.2%)
Brito et al., 2012^31^	Mexico City, Mexico	2008	Lesbian, gay, bisexual and transgender people	Convenience; cross-sectional	Lifetime experience of physical or sexual aggression	All groups: 823	Physical aggression: 149 (18.1%); Sexual: 75 (9.1%)
						Homosexual male: 467	Physical aggression: 89 (19.1%) Sexual violence: 62 (13.3%)
						Homosexual female: 152	Physical aggression: 19 (12.5%) Sexual violence: 11 (7.5%)
						Bisexual male: 60	Physical aggression: 10 (16.7%) Sexual violence: 5 (8.6%)
						Bisexual female: 69	Physical aggression: 4 (5.8%) Sexual violence: 4 (5.8%)
						Transgender: 71	Physical aggression: 27 (38.0%) Sexual violence: 15 (21.1%)
Guasp, 2012^53^	United Kingdom of Great Britain and Northern Ireland	2011–2012	Lesbian, gay and bisexual people (12–19 years old)	Convenience; cross-sectional	Lifetime experience of homophobic bullying in and around school: physical abuse or sexual assault	1614	Physical abuse: N/A (16%) Sexual assault: N/A (3%)
Iosa et al., 2012^60^	Córdoba, Argentina	2010	Lesbian, gay, bisexual and transgender people	Convenience; cross-sectional	Lifetime experience of physical aggression or sexual violence	All groups: 347	Physical violence: 81 (23.3%) Sexual violence: 29 (8.4%)
						Gay: 174	Physical violence: 42 (24.1%) Sexual violence: 8 (4.6%)
						Lesbian: 95	Physical violence: 13 (13.7%) Sexual violence: 7 (7.4%)
						Bisexual: 44	Physical violence: 6 (13.6%) Sexual violence: 4 (9.1%)
						Transgender: 34	Physical violence: 20 (58.8%) Sexual violence: 10 (29.4%)
Kosciw et al., 2012^69^	USA	2011	Lesbian, gay, bisexual and transgender youth (13–20 years old)	Convenience; cross-sectional	In the past 12 months been physically assaulted at school	8584	Physical assault based on sexual orientation: N/A (18.3%); gender expression: N/A (12.4%)
Leonard et al., 2012^74^	Australia	2011	Sexual and gender minorities	Convenience; cross-sectional	In the past 12 months, been sexually assaulted or physically attacked with a weapon	All groups: 3835	Physical attack: N/A (1.8%) Sexual assault: N/A (2.9%)
						Male: 1701	Physical attack: N/A (2.2%) Sexual assault: N/A (2.3%)
						Female: 1849	Physical attack: N/A (1.3%) Sexual assault: N/A (3.1%)
						Transgender male: 47	Physical attack: N/A (0.0%) Sexual assault: N/A (0.0%)
						Transgender female: 122	Physical attack: N/A (2.5%) Sexual assault: N/A (6.8%)
						Other gender identity: 116	Physical attack: N/A (6.2%) Sexual assault: N/A (4.5%)
Levitt et al., 2012^75^	USA and Canada	N/A	Sexual minority women (non-androgynous identity)	Convenience; cross-sectional	Lifetime experience of throw object, physical attack or sexual assault	909	Object thrown: 29 (3.7%) Physical attack: 36 (4.6%) Sexual assault: 24 (3.0%)
McNeil et al., 2012^79^	United Kingdom of Great Britain and Northern Ireland	2012	Transgender people	Convenience; cross-sectional	In past week, past year, past 1–10 years past 10 years or ever been hit or beaten up, sexually assaulted or raped	889	Hit or beaten up in past week: N/A (0%); past 12 months: N/A (5%); past 1–10 years (10%); > 10 years: N/A (5%); lifetime N/A (19%) Sexual assault in past week: N/A (0%); past 12 months: N/A (4%); past 1–10 years: N/A (7%); > 10 years: N/A (2%); lifetime (14%) Rape in past week: N/A (0%); past 12 months: N/A (2%); past 1–10 years: N/A (3%); > 10 years: N/A (2%); lifetime N/A (6%)
Motmans et al., 2012^82^	Belgium	2012	Transgender people	Convenience; cross-sectional	Lifetime experience of physical violence or sexual violence	260	Physical violence: N/A (27%) Sexual violence: N/A (32%)
Oogachaga 2012^86^	Singapore	2012	Lesbian, gay, bisexual, transgender and queer people	Convenience; cross-sectional	Lifetime experience of physical attack or controls on movements	Same-sex-attracted male: 272	Physical violence: N/A (6.8%)
						Same-sex-attracted female: 134	Physical violence: N/A (3.7%)
						Male-to-female transgender: 18	Physical violence: N/A (22.2%)
						Female-to-male transgender: 14	Physical violence: N/A (14.3%)
Testa et al., 2012^99^	Virginia, USA	2005–2006	Transgender people (transitioning)	Convenience; cross-sectional	Lifetime experience of physical or sexual violence	271	Physical violence: N/A (37.1%) Sexual violence: for any reason: N/A (23.7%)
Chamberland et al., 2013^38^	Québec, Canada	2009	Lesbian, gay, bisexual and queer people (3rd–5th year of secondary school)	Venue-based; cross-sectional	Since beginning of the school year (6–8 months) been pushed or hit or having objects thrown	All groups: 213	Physical violence: 39 (18.3%)
de Sousa et al., 2013^47^	Recife, Brazil	2008–2009	Male-to-female transgender people	Response driven; cross-sectional	Lifetime experience of physical aggression or sexual violence	110	Physical aggression: 75 (68.2%) Sexual violence: 54 (49.1%)
Pelullo et al., 2013^90^	Naples, Italy	2011	Lesbian, gay and bisexual people	Convenience; cross-sectional	Ever experienced episodes of victimization: physical or sexual violence	1000	Physical or sexual violence in past 12 months: 18 (1.8%); lifetime: 74 (7.4%)
Aho et al., 2014^24^	Abidjan, Côte d'Ivoire	2011–2012	Men who have sex with men	Cross-sectional; Respondent Driven Sampling	History of coerced sex or physical abuse	603	Physical abuse: N/A (8.5%; 95% CI: 5.5–11.4)
Boza et al., 2014^29^	Australia	2012	Transgender people	Convenience; cross-sectional	Lifetime experience of physical or sexual violence	255	Objects thrown: 18 (7.4%) Assault without a weapon: 25 (10.3%) Assault with a weapon: 7 (2.9%); Sexual assault: 17 (7.0%); Attempted rape: 4 (1.6%); Rape: 8 (3.3%)
de Deus 2014^46^	São Paulo, Brazil	2011–2012	Men who have sex with men	Time-location; cross-sectional	Lifetime experience of physical aggression or sexual violence	1215	Physical aggression: 268 (22.1%)^d^ Sexual violence: 86/1214 (7.1%)^d^
Herrick et al., 2014^55^	Los Angeles, USA	2005–2006	Men who have sex with men (18–24 years old)	Venue-day-time; longitudinal	Lifetime experience of physical victimization	470	Physical victimization: 107 (22.8%)
Ivanković et al., 2014^61^	Croatia	2011–2013	Men who have sex with men (18–50 years old)	Convenience; cross-sectional	Lifetime experience of physical abuse (hit or beaten)	507	Hit: N/A (23.4%)^e^ Beaten: N/A (10.6%)^e^
Kosciw et al., 2014^70^	USA	2013	Lesbian, gay, bisexual and transgender youth (13–21 years old)	Convenience; cross-sectional	In the past 12 months been physically assaulted at school	7898	Physical assault based on: sexual orientation: N/A (16.5%); gender expression: N/A (11.4%)
Lea et al., 2014^73^	Sydney, Australia	2010	Lesbian, gay and bisexual people (18–25 years old)	Convenience; cross-sectional	Ever or in the past 12 months been physically abused	Gay: 301	Physical abuse in past 12 months: 27 (9.0%); lifetime: 87 (28.9%)
						Bisexual male: 17	Physical abuse in past 12 months: 0 (0.0%); lifetime: 2 (11.8%)
						Lesbian: 146	Physical abuse in past 12 months: 9 (6.2%); lifetime: 35 (24.0%)
						Bisexual female: 108	Physical abuse in past 12 months: 2 (1.9%); lifetime: 21 (19.4%)
Mereish et al., 2014^80^	New England, USA	2001–2003	Sexual and gender minorities	Convenience; cross-sectional	Lifetime experience of physical attack	1457	Physical violence: 246 (16.9%)
Nuttbrock et al., 2014^85^	New York, USA	2004–2007	Transgender women	Convenience; longitudinal	In the last 6 months been physically abused	230	Physical abuse: N/A (10.0%)
Scruton, 2014^96^	Canada	2013–2014	Transgender people	Convenience; cross-sectional	Lifetime experience of physical violence or sexual assault	267	Physical violence: N/A (22%) Sexual assault: N/A (19%)
Smith et al., 2014^97^	Australia	N/A	Transgender and gender variant people (age 14–25 years)	Convenience; cross-sectional	Lifetime experience of physical abuse	189	Physical violence: 38 (20.1%)
Strizzi et al., 2014^98^	Spain and USA	N/A	Lesbian, gay, bisexual and queer people	Convenience; cross-sectional	In the past year had object thrown. Lifetime experience of physical or sexual assault	USA: 83	Object thrown: N/A (14%) Physical assault: N/A (6.0%) Sexual assault: N/A (8.7%)
						Spain: 157	Object thrown: N/A (10%) Physical assault: N/A (6%) Sexual assault: 0 (0%)
Bauer et al., 2015^28^	Ontario, Canada	2009–2010	Transgender and gender variant people (age 14–25 years)	Cross-sectional; respondent-driven Sampling	Lifetime experience of physical or sexual harassment and violence	380	Physical or sexual assault: N/A (21.2%; 95% CI: 15.0–27.3%)
Burks et al., 2015^32^	Houston, USA	2015	Lesbian, gay, bisexual and transgender people	Convenience; cross-sectional	Lifetime experience of physical attack or sexual assault	All groups: 336	Physical attack: 61 (18.2%) Sexual assault: 34 (10.1%)
Ferlatte et al., 2015^49^	British Columbia, Canada	2011–2012	Gay and bisexual people	Convenience; cross-sectional	Lifetime experience of physical and sexual violence (unwanted sex)	8382	Physical violence: 1044 (12.5%) Sexual violence: 985 (11.8%)
Goldbach et al., 2015^51^	USA	2000	Lesbian, gay and bisexual people (12–18 years old)	Convenience; cross-sectional	Lifetime experience of beating, physical violence or having object thrown	1911	Beaten: 167 (10%) Physical violence: 421 (25%) Object thrown: 305 (18%)
Barrientos et al., 2016^27^	Arica, Valparaiso, and Santiago, Chile	2011	Men who have sex with men and male-to-female transgender people	Cross-sectional; respondent-driven sampling (men who have sex with men) and snowball (transgender people)	Lifetime experience of physical or sexual aggression or violent assault (robbery with violence)	Gay: 325	Physical aggression: 54 (16.6%) Sexual aggression: 37 (11.5%) Violent assault: 44 (13.7%)
						Transgender: 112	Physical aggression: 68 (61.3%) Sexual aggression: 45 (40.5%) Violent assault: 59 (53.2%)
D’haese et al., 2016^45^	Flemish Community, Belgium	2013	Lesbian, gay and bisexual people	Convenience; cross-sectional	Lifetime experience of physical violence	All groups: 1402	Physical violence: 436 (31.1%)
						Male: 916	Physical violence: 318 (34.7%)
						Female: 486	Physical violence: 118 (24.3%)
Kramer et al., 2016^71^	38 European countries	2011	Men who have sex with men	Convenience; cross-sectional	In the past 12 months been punched, hit, kicked or beaten	91 477	Punched, hit, kicked or beaten: N/A (2.5%)^f^
Rodriguez-Madera et al., 2016^93^	San Juan, Puerto Rico	2011–2013	Transgender women	Respondent-driven sampling; cross-sectional	Lifetime experience of physical or sexual violence	59	Physical violence: 16 (weighted percentage: 25%) Sexual violence: 8 (weighted percentage: 16%)

CI: confidence interval; N/A: data not available; SD: standard deviation; USA: United States of America.

^a^ Definitions of terms were based on the 2015 Joint United Nations Programme on HIV and AIDS terminology guidelines (Box 1).^23^

^b^ We only report violence perceived by the victim to be based on sexual orientation or gender identity/expression. Specific descriptions and definitions of physical and sexual violence that were used to elicit participants’ responses varied across studies (Box 3).

^c^ Number of cases are not reported in all articles, notably for respondent-driven sampling where different weights are given to different participants.

^d^ Data from a presentation of the study.

^e^ Data provided by the author.

^f^ Data from the technical report of the study.

Twenty-six studies included all sexual and gender minorities, of which eight were exclusively high-school students. Thirteen included homosexual and bisexual participants, of which five focused only on younger participants (maximum age 25 years) and one only on older participants (minimum age 60 years). Thirteen studies included homosexual or bisexual men and of these 8 targeted specific groups: bisexual men (4 studies); Latino men (1 study); homosexual or bisexual men aged < 29 years (1 study); seronegative homosexual or bisexual men aged 15‒30 years (1 study); and non-white seropositive homosexual or bisexual men aged 13‒24 years (1 study). Homosexual or bisexual women were exclusively sampled in four studies, of which three targeted specific groups: bisexual women (2 studies) and sexual minority women of non-androgynous identity (1 study). One study sampled young people who experienced same-sex attraction and another included the same study group together with young people who questioned their gender. One study sampled homosexual or bisexual men and male-to-female transgender people. Fifteen studies were of transgender people, of which five studies were specific groups only: male-to-female transgender people (3 studies), male-to-female individuals who were sex workers (1 study); and transitioning transgender people (1 study).

The descriptions and definitions of physical and sexual violence motivated by perception of sexual orientation and gender identity that were used to elicit participants’ responses varied across studies (Box 3). These included the victim’s perception of the motivation of the violence and the types of violence experienced. A few studies used lists of specific violent acts or a combination of actions or scales with multiple items to measure experiences of different kinds of physical violence. Similarly, in the category of sexual violence several different definitions were used in different studies.

Box 3 **Terminology used in studies included in the systematic review of physical and sexual violence motivated by perception of sexual orientation and gender identity**

A wide range of descriptions and definitions of violence were used to elicit responses in the included studies:

**Motivation for the violence**

The victim’s perception of the motivation of the violence was variously defined as: “because you’re lesbian/gay/bisexual (or someone thought you were)”, “because somebody thought or knew you were gay?”, “because of/based on/attributed to (perceived) sexual orientation”, “on the grounds of homosexuality”, “related to MSM-status,” “because of/based on sexuality”, “because of your sexual identity (or sexual preferences)”, “experienced lesbophobic situation”, “because someone knew or presumed you are attracted to men?”, “based on sexual orientation and gender identity”, “on the basis of gender issues”, “for being gay or being perceived as effeminate”, “related to their sexual orientation, how they express their gender”, “due to gender stereotype transgression”, “due to being trans(gender)”, “(thought it was) because of gender identity (or gender presentation)”, “because you’re trans or because of your gender expression”, “for being transgender or effeminate”, “because you’re lesbian/gay/bisexual/transgender”, “transgender status, gender identity or expression”, “because of the status as a transgender person”, “because of transgender identity or background”, “due to being queer”, “an incident that you felt was homophobic (or transphobic)”, “an anti-lesbian/gay incident”, “heterosexist violence and harassment because of sexuality or gender identity”.

**Types of physical violence**

Different terms for physical violence were used in different articles. Some used “physical violence”, others combined “physical” with “attack”, “assault”, “victimization”, “abuse”, “aggression”, “mistreatment” or “injury”.

One article used a longer definition: “the intentional use of physical force with the potential for causing death, disability, injury, or harm; some examples: scratching, pushing, shoving, throwing, grabbing, biting, choking, shaking, slapping, punching”. Another used the term “criminal victimization”, including specific incidents of physical violence: “experience of a crime against their person (hit, beaten, physically attacked, sexually assaulted)....”.

Similarly, most articles included specific violent acts or a combination of actions: “thrown some object”, “hit”, “knocked down”, “injured with some weapon”, “punched”, “kicked”, “beaten”, “hurt with a knife, gun, bat, or some other weapon”, “assault/robbery with violence”, “assault with a weapon, assault without a weapon”, “grievous bodily harm”, “attempted murder” and “violent assault”.

One article used an extensive scale to measure physical violence. Physical violence was surveyed making use of 11 items, ranging from “an object was thrown at me”, “I was being pushed or pulled”, “someone hit me with his or her hand” to “someone tried to strangle or suffocate me”.

**Types of sexual violence**

Several different terms were used in different articles to define sexual violence: “sexual violence”, “sexual assault”, “rape”, “sexual aggression”, “sexual victimization”, “sexual abuse” and “other sexual violence”.

In some publications definitions for sexual violence or similar concepts were applied: “ever been forced to engage in unwanted sexual activity”, “any sexual act that is perpetrated against someone’s will; some examples: completed non-consensual sex act, an attempted non-consensual sex act, abusive sexual contact and non-contact sexual abuse”, “sexual aggression: sexually molested and/or forced to have sexual relations with penetration” and “sexual victimization: ever been sexually abused or raped”.

MSM: men who have sex with men; trans: transgender.

Fifty-seven studies asked about experiences of violence ever in the respondent’s lifetime. Six studies specified experiences over certain stages of the lifetime: from 13 years old (1 study); ages 6–10 years, 11–17 years and 18+ years (1 study); 1 year ago, 1‒10 years ago and > 10 years ago (1 study); age 12‒18 years and 18+ years (2 studies); or childhood versus adulthood (1 study). Other studies asked about experiences over specific time periods: 5 years (1 study); 2 years (1 study); 12 months (21 studies); 6 months (3 studies); or 1 month (2 studies). Ten studies asked about experiences in school: past year in school (7 studies); ever in school (1 study); during high-school years (1 study); and since the beginning of the school year (1 study). Some articles measured violence experienced both over the lifetime and over certain periods.

### Prevalence of violence

**Fig. 2:**
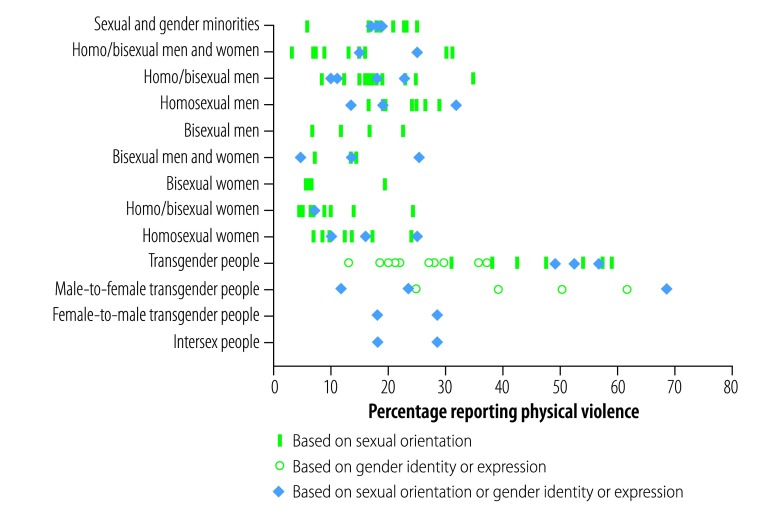
Lifetime prevalence of physical violence motivated by perception of sexual orientation and gender identity, by perceived motivation for the attack

Notes: Based on 51 studies.^24^^–^^27^^,^^30^^–^^32^^,^^34^^–^^37^^,^^39^^–^^42^^,^^44^^–^^58^^,^^60^^,^^62^^,^^63^^,^^72^^,^^73^^,^^75^^,^^80^^–^^84^^,^^86^^,^^88^^,^^91^^–^^93^^,^^96^^–^^100^ Six of the 57 studies were not included because they measured a particular form of physical violence, such as being hit or beaten. Data are presented from the smallest subgroup in each study.

A total of 57 studies provided data on the lifetime prevalence of any kind of physical violence motivated by perception of sexual orientation and gender identity (Table 2). Fig. 2 summarizes the data for 51 studies, according to the different populations and the attacker’s motivation as perceived by the victim (sexual orientation, gender identity or both). In 14 studies where all sexual and gender minorities were taken together the prevalence ranged from 6% in a study of 240 people^98^ to 25.0% (49/196).^26^ When transgender people were not included (11 studies) the figures ranged from 3.3% (24/761)^100^ to 31.1% (436/1402).^45^ In homosexual or bisexual men (29 studies), the prevalence was between 8.5% in a study of 603 people^24^ and 34.7% (318/916),^45^ although when only bisexual men were included (4 studies), the prevalence was no higher than 22.6% (12/53).^37^ A similar tendency was observed in homosexual or bisexual women (21 studies), with a prevalence range from 4.6% in a study including 909 individuals^75^ to 25.1% (42/167 people),^30^ and a lower prevalence when bisexual women only were included (4 studies). For transgender people prevalence (28 studies) ranged from 11.8% of a sample size of 34^91^ to 68.2% (75/110 people).^47^

There was no pattern of prevalence for the perceived motivation of the violence (sexual orientation, gender identity or both). The lifetime prevalence of violence in younger aged samples did not seem to be lower (Table 2).

Seven studies reported data specifically on the lifetime prevalence of being punched, kicked, hit or beaten up. In homosexual or bisexual men and women the lowest value was 10% in a study sampling a total of 1911 people,^51^ and the highest value was 15.6% (62/416 people).^42^ In studies sampling only men the prevalence peaked at 23.4% (of a total sample of 506).^61^

In 10 studies the researchers asked homosexual or bisexual women specifically about having objects thrown at them motivated by homophobia or transphobia. The prevalence ranged from 3.7% (in a study of 909 sexual minority women)^75^ up to 35.0% (65/186 lesbian, gay and bisexual people).^62^ Among transgender people, values ranged from 7.4% (in a study of 255 people)^29^ to 17.4% (70/402).^78^

### Prevalence of sexual violence

**Fig. 3:**
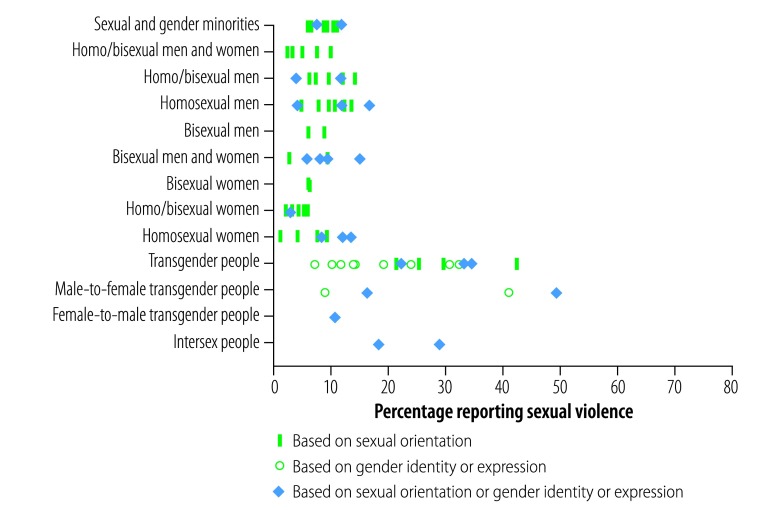
Lifetime prevalence of sexual violence motivated by perception of sexual orientation and gender identity, by perceived motivation for the attack

Notes: Based on 33 studies.^25^^–^^27^^,^^29^^–^^32^^,^^34^^–^^37^^,^^41^^–^^44^^,^^46^^,^^47^^,^^49^^,^^50^^,^^53^^,^^60^^,^^62^^,^^63^^,^^75^^,^^78^^,^^79^^,^^82^^,^^91^^–^^93^^,^^96^^,^^98^^,^^99^ Data are presented from the smallest subgroup in each study.

Fig. 3 shows the data from 33 studies reporting lifetime prevalence of any kind of sexual violence motivated by perception of sexual orientation and gender identity. The prevalence ranged from 5.6% (28/504 people)^35^ to 11.4% (55/484) for all sexual and gender minority groups (12 studies),^50^ and from 2.1% (8/377)^92^ to 9.7% (18/186)^62^ when only homosexual or bisexual men and women were considered (5 studies). The prevalence in homosexual or bisexual men (17 studies) ranged from 3.7% in a study sampling 3429 people^91^ to 16.5% (69/419 people).^30^ This was slightly higher than in studies of homosexual or bisexual women (8 studies), where it ranged from 1.0% (1/102 people)^34^ to 13.2% (14/106).^50^ When bisexual people were disaggregated (10 studies), the prevalence ranged from 2.4% (1/41 people)^34^ to 14.7% (24/95).^30^ Between 7.0% (in a study of 255 people)^29^ and 49.1% (54/110 people)^47^ of transgender people reported sexual violence (22 studies).

Six studies reported specifically on rape (Table 2). Among homosexual or bisexual men and women between 0.3% (6/1740 people)^33^ and 10.0% (of 264 people)^88^ reported ever being raped due to their sexual orientation or gender identity, with figures for men being higher than those for women. The prevalence of rape for transgender people ranged from 3.3% (in a study sampling 255 people)^29^ to 9.9% (25/253 people).^41^

## Discussion

Our review found a high prevalence of physical and sexual violence motivated by perception of sexual orientation and gender identity experienced by sexual and gender minorities, particularly among transgender people. These values suggest that such violence accounts for a large part of all the violence encountered by sexual and gender minorities. Nevertheless, it remains to be researched whether such violence explains the higher prevalence of violence against sexual and gender minorities in comparison with the rest of the population. The higher prevalence in transgender people might be partly explained by a higher risk of being involved in sex work.^101^

Violence motivated by perception of sexual orientation and gender identity might not be confined to a minority population. Recent research identified distinct populations on the sexual orientation continuum who identify as mostly heterosexual with a small degree of same-sex sexual or romantic attraction, including occasionally having sexual relations with someone from the same sex.^102^ Although we found no publications on this population, earlier research has shown they were 1.47 times more likely than heterosexuals to report experiences of childhood victimization by adults. This elevated proportion is similar to those found among homosexual or bisexual men and women compared to heterosexuals, which might be explained by gender non-conformity in childhood.^103^ Moreover, people who do not belong to a sexual or gender minority, have also reported being victims of violence motivated by perception of sexual orientation and gender identity.^104^

A review of systematic reviews showed that sexual and gender minorities are highly burdened by human immunodeficiency virus infection, sexually transmitted infections, sexually transmitted infection-related cancers, mental health conditions and violent experiences.^105^ We suggest further research into the associations of violence motivated by perception of sexual orientation and gender identity with adverse health and social outcomes, including criminalization. This includes the effect of what has been termed syndemic vulnerability^106^ or the synergistic interaction between health conditions, exacerbated under circumstances of structural and political adversity.

If we want to eradicate violence motivated by perception of sexual orientation and gender identity, we must identify the mechanisms and motivations of such violence. The perpetrators are often male and although violence is not necessarily a part of men’s dominant position in society (hegemonic masculinity), the two are often linked. In many parts of the world, women are perceived as inferior and therefore both femininity and homosexuality are denigrated and discredited.^107^ Physical or sexual force and threats are ways to achieve control, including punishment of perceived acts of resistance to or transgression of gender norms and behaviours.^108^ Although same-sex attraction and gender nonconformity can negatively affect the personal relations of individuals with their peers,^109^ some authors believe that sexual and gender minorities are mainly attacked because they defy gender stereotypes.^87^ This has prompted calls for the elimination of the dichotomist gender characterization.^87^

The quality of our data was relatively poor due to a lack of standardized measures and sometimes small and non-randomized samples. The evidence base needs to be strengthened. More and better research on the prevalence and adverse outcomes of violence motivated by perception of sexual orientation and gender identity is needed across many different geographical and cultural settings (especially outside the USA) and different socioeconomic and age groups. Community organizations should be empowered to add scientific value to their existing efforts to map such violence. A consensus is needed on definitions and measures of violence motivated by perception of sexual orientation and gender identity and how to operationalize them to allow for comparisons across studies.

Some limitations of this review are that most studies used a non-probability sample, mostly a convenience sample, and provided little information on the representativeness of the sample, the potential impact of non-participation, or the study power. The reliability and comparability of studies were limited, as it was not possible to compare between countries, regions or cultural backgrounds. The studies relied on the participants’ self-reports to determine whether they had been a victim of violence and whether that violence was motivated by their sexual orientation and gender identity. Without increased understanding of respondents’ narratives about violence and its motives, research in this field will be vulnerable to criticism.^110^

Despite these limitations, our review shows that high proportions of sexual and gender minorities experienced physical and sexual violence, motivated by perception of sexual orientation and gender identity, which might have an effect on their health and well-being. National violence prevention policies and interventions should include such violence, integrating it into national health surveys and health promotion efforts and improve data collection and reporting of incidents.
